# Perspectives of digital agriculture in diverse types of livestock supply chain systems. Making sense of uses and benefits

**DOI:** 10.3389/fvets.2022.992882

**Published:** 2022-12-02

**Authors:** Derek Baker, Elizabeth L. Jackson, Simon Cook

**Affiliations:** ^1^Centre for Agribusiness, University of New England, Armidale, NSW, Australia; ^2^Food Agility CRC, Sydney, NSW, Australia; ^3^School of Management and Marketing, Curtin University, Bentley, WA, Australia; ^4^College of Science, Health, Engineering and Education, Murdoch University, Murdoch, WA, Australia

**Keywords:** digital agriculture, livestock systems, supply chain, product quality, adoption

## Abstract

Digital technology is being introduced to global agriculture in a wide variety of forms that are collectively known as digital agriculture. In this paper we provide opportunities and value propositions of how this is occurring in livestock production systems, with a consistent emphasis on technology relating to animal health, animal welfare, and product quality for value creation. This is achieved by organizing individual accounts of digital agriculture in livestock systems according to four broad types—commodity-based; value seeking; subsistence and nature-based. Each type presents contrasting modes of value creation in downstream processing; as well as from the perspective of One Health. The ideal result of digital technology adoption is an equitable and substantial diversification of supply chains, increased monetization of animal product quality, and more sensitive management to meet customer demands and environmental threats. Such changes have a significance beyond the immediate value generated because they indicate endogenous growth in livestock systems, and may concern externalities imposed by the pursuit of purely commercial ends.

## Introduction

Recent years have resulted in major changes to personal and professional lives through the introduction of digital technologies. Readers of this paper will have experienced some of these: in finance, transport, retailing, construction and other sectors (1). High expectations are placed on the potential of digital technologies to help agriculture meet the challenges it faces in the coming decades (2–6). Digital agriculture is a relatively recent term, appearing since 2015, to describe a wide range of applications of digital technology within agriculture both on the farm and along the supply chain, as well as in the realms of agriculture's delivery of nature services and public goods; Cook et al. (7) provided a four-part categorization of digital agricultural technologies to broadly define their scope: (1) Data: Sensing the system, (2) Control: Responding to insight, (3) Modeling: Working out what complex multi-sensor data means and (4) Networking and Communication: Increasing the flow of data and insight.

The agricultural sector was assessed by McKinsey & Co (8) in 2015 to be the least digitized of 23 in the US economy and 2 years later to be the least digitized of all Australian sectors (9). However, investment in digital agri-food technology has grown strongly over recent years, even during the coronavirus pandemic. Global venture capital in such activities grew to about $51 billion in 2021 and is entering a phase of maturation (10). This paper addresses the innovations taken by these technical and financial developments in livestock systems.

At the same time as the excitement of investors emerges, Shiller (11) added that investor sentiment is driven by narratives that precede certainty. Investors who risk funds—and scientists who risk valuable years of their careers—often pursue these changes in order to separate hype from reality and understand the nature of digital agriculture change. From a review of many different examples of digital agriculture, Cook et al. (7) concluded that major investment in digital agriculture was indeed likely—if for no other reason than simple arbitrage—but that the drivers of change and its direction remained confused among innovation users, creators and investors. We propose that the diverse technical, financial and behavioral nature of these drivers is a consequence of the different needs and value propositions exhibited across livestock system types. In the current paper we map these value propositions to an array of livestock systems, grouped for convenience into some (perhaps overlapping) functionalities and contexts.

The scope of change portrayed by many writers on digital agriculture is broad, and often focused on technologies rather than the problems or opportunities to which technology is targeted. Shepherd et al. [(12). p. 5,084] defined digital agriculture as “the use of detailed digital information to guide decisions along the agricultural value chain.” Many works reviewed for this paper were restricted to pre-defined sectors of change processes, such as implications for sustainable production (13, 14), the social implications (15), or big data operations (16–21), and the likely impacts in specific areas, such as Australia or other specific country and agricultural development globally (2, 22–24). From these we determined that it was helpful to understand the function of food systems (25) in order to organize the many changes offered by digital agriculture, with particular reference to livestock production systems. We observed that unlike its predecessor, precision agriculture, digital agriculture operated throughout food systems, in the domains of production, processing, marketing, capitals and governance (7), even as the systems morphed to respond to opportunities and threats (26).

The potential for change through digital agricultural and its associated technology might be vast, but change will be realized through value propositions that define how technology use creates value and who acquires it (27). The issue relates to characteristic patterns of technology uses in different sectors of the economy (28). We find the detailed classification developed for manufacturing to be helpful (29) because it describes the range of organizations and behaviors likely to be found in food systems, rather than the perhaps over-simplified inventory approach of Manyika et al. (8) which overlooks the diversity of technology use—including technology embedded in agricultural production systems (30).

In what follows, we draw on food systems thinking (31) to capture the diversity of opportunities and value propositions available to the livestock production sector from digital technologies with a consistent focus on animal welfare for value creation in supply chain systems. Food systems thinking draws together technical, economic and social influences and processes, to provide a multi-objective interpretation of change such as technology adoption. A full understanding of livestock's contributions to the environment, to One Health, and to subsistence households' livelihood are examples of objectives which have been brought to the fore as systems thinking and methods have allowed these to be expanded into something more holistic. The links between animal welfare, feed intake, various behavioral variables, sustainability and productivity have been exploited using digital tools (14, 32–34). The tools comprise technology, with appropriate algorithms, to manipulate data generated in various sensor environments. Understanding how actors acquire (or fail to acquire) value from such technology is important to determine whether or not system change is likely. This is vastly different for the range of livestock systems operating in various regions of the world. From this, we attempt to organize the vast range of applications for digital technologies within the fields of animal health and welfare and the implications of these for supply chains. Our purpose is to enable readers to understand the dynamics of adoption and the emerging scientific fields to support them.

## Livestock system types

Livestock systems classification is a mature field of knowledge with seminal enquiry emerging in the 1980s [for example (35, 36)] with further contributions being made in more recent times [for example (37, 38)]. Given that livestock production systems are highly dependent upon downstream value creation (i.e., value creation occurs when commodity products like meat, milk and wool are differentiated at the processing, manufacturing and retail end of the supply chain), Pavitt's (29) classification was helpful in considering livestock systems from a value perspective borne from patterns of technical change, rather than the traditional production perspective. Unlike current knowledge on animal-focused classification schemes, the essence of Pavitt's (29) classification is describing and explaining sectoral-levels of technical change. Pavitt's (29) three-part taxonomy created the foundation of thinking about technical change at scale. It provides a framework for understanding sources and directions of how firms change in the context of diversification behavior and how technical skills and advantages are created. Most pertinent to agri-food supply chains, which are arguably characterized as commodity-based, is the acknowledged nexus between technology and industry structure.

Table 1 presents four illustrative livestock system classes which we discuss. The basis for this grouping is the taxonomy offered to explain the contrasting forms of adoption of technology in industry at the sectore-level (29), to which we apply observations from our experience of livestock sectors in Australia, the UK and elsewhere. We also postulate seven forms of benefit available from digital technologies: these provide a spectrum from private goods such as reduced production costs, to shared benefits throughout the supply chain such as enhanced disease surveillance and sales price increases due to product attribute differentiation, and onwards to public goods such as resource conservation. The body of Table 1 lists digital products and applications, and its bottom row lists the features of each system that enables, at least potentially, value generation from the technology. The right-hand column of the table lists, for each form of value, the enabling factors for livestock production and supply chains. As we shall see, not all systems offer up these features.

**Table 1:** General function of digital technologies for animal health, animal welfare and product quality in systems for the four classes of livestock.

		**Class of livestock system**				**Enabling factors for each system**
		**Commodity producers**	**Value seekers**	**Subsistence farmers**	**Nature-based systems**	
Forms of benefit from adoption of digital technologies	Production cost savings	• Sensors and data enable more precise management of production variation e.g., animals exhibiting natural/unnatural behaviors	• Data and process control enables price gains	• Low cost, wide-area distributed data supports efficiency gains	• Labor deployment away from information-intensive tasks	Cost, robustness and functionality of sensors. Productivity metrics
	Data assets for compliance or marketing as product attributes	• Data from sensors certify compliance • Data supports product streaming	• Data and communication tech (e.g., block chain) certify product provenance. Modeling to certify quality attribute.	• Real-time monitoring of disease status • Tracking of produce within value chains to improve biosecurity	• Provenance and production systems certifiable • Monitoring & modeling of land /water resources to provide whole-of value chain perspective	Data capture, metrics, control and integrity
	Data integration and aggregation for commercial use	• Targeted quality • Scheduling of production batches • Benchmarking performance	• Targeting consumers' willingness to pay • Connection to supply chain partners • Animal welfare in extensive grazing systems monitored • Product selective processing (e.g., Dual Energy X-Ray Absorptiometry) • Biosecurity	• Animal ownership tracking. • Cloud-based animal identification systems for smallholders • Disease forecasting	• Sensors provide data on animal location • Remote sensing identifies invasive species control • Monitoring product flow improved biosecurity • Long term climate adaptation	Connectivity Data ownership and governance Data interoperability
	Risk management	• Climate predictions • Stocking rates' manipulation • Seasonal timing of production and sales • Biosecurity monitoring	• Informed marketing choices • Feed and water management e.g., video surveillance of intake	• Enabling choices between specialization and diversification • Feed management	• Remote sensing and modeling for index-based insurance	Supply chain co-ordination for risk management
	Enhanced resource management	• Breeding/genetics • Soil management • Water accounting • Carbon finance • Life Cycle Analysis	• Mapping resources to data assets e.g., Real-time feed monitoring	• Monitoring of key sustainability variables	• Whole-of-system modeling for resilience, complementing local knowledge and replacing dedicated labor input • Farm-scale data for community-based management	Business model for sharing remote sensing data
	Enhanced delivery of public goods	• Disease monitoring e.g., video, audio, thermal analysis • Animal husbandry compliance	• Compliance as quality attributes e.g., video behavioral monitoring & thermal analysis of health attributes	• Disease surveillance	• Surveillance on resource quality	Data integrity Data interoperability
	Enabling features of each system	• Scale of operation relative to investments	• Supply chain partnerships • Scope of operations	• Collective action on data acquisition • Public-private partnerships	• Willingness to free up labor • Integration of multiple international data and analytics systems	

We present “commodity producers” as medium to large scale livestock operations serving mature and demanding markets for large volumes of product of consistent quality. Red meat grazing operations in this category are highly seasonal and face associated risks; feedlots and monogastric operations focus on throughput and the risks of price movements. Both feature large scale operations, with cost structures favoring ever more scale. Feed supplies and prices fluctuate, and animal disease is a constant threat managed at significant cost. Information has traditionally been expensive to collect, and rewards difficult to capture. Costs and productivity dominate management objectives, and key metrics include stocking rates, resource efficiency, and timing of operations. Marketing initiatives are led from downstream in the supply chain and are manifest as compliance requirements: particularly for animal welfare and production methods that remain in the responsibility of farmers. Management efficacy is sufficient in that disease control, along with animal welfare, remains a private good in many economies. Enterprise size is sufficient to on-farm water management and emerging needs of farms like mobilizing carbon sequestration.

Our “value seekers” type of livestock operation may be small and mixed with other farm operations, and pursues cost savings on the basis of targeted inputs, and price advantages due to differentiation and the targeting of customers along the supply chain through to consumers. This requires not only collection of information but its transmission and interpretation: first about products' physical attributes but increasingly about provenance and process. The higher value of products invites investment at processing and retail stages of the supply chain, and capturing value features partnerships of various types including data integration. Examples include dairy production targeting consumers seeking consumption and use experiences based on dis-assembly and re-assembly of milk components, and a vertically-coordinated approach to value addition and pricing.

“Subsistence farmers,” as we present them, operate diverse and generally small scale livestock enterprises with products either for family consumption (eggs and milk) or for sale to fund household necessities and social activities. Risks are encountered such as seasonality, market vagaries, and plant and animal disease. Operators are diverse, usually with multiple sources of income, but some threats—notably animal disease—are felt by all and lend themselves to a common response. Readers should note that our classification centres on value and the means of its delivery. Conventional systems' classifications such as “pastoralist” might be invoked for our subsistence type: but the inclusion of scale of operation, shared but intensive resource use, critical timing of operations, and linkage to product and asset markets all mean that we treat some pastoralists as commodity producers, others as nature based, and only some pastoralists as subsistence farmers.

“Nature based systems” are spatially extensive, highly sensitive to climate and disease, and labor intensive livestock systems. Importantly, these systems rely little on purchased or scheduled inputs: communal grazing systems provide an example. Overall management objectives centre on resilience, with animals commonly the store of value as accumulated savings. All operators face the same risks, mostly derived from external factors, and this enables approaches to risk management based on entire systems and landscapes with shared costs. The specification of labor intensiveness refers to these systems' lack of purchased inputs and mechanization: the assets—animals and other assets—are employed jointly with labor which is not generally available for other uses. Tasks such as herding and tending lend themselves strongly to labor saving digital technologies, as we shall see.

An important point to conclude on is that these classification systems of digital agriculture are not limited to corporate agriculture or big business. There is plenty of recent evidence to confirm that digital agriculture is accessible to disparate geographies, demographies, and societies (39–41) so it therefore relatively ubiquitous in democratizing access to technical change. Examples of this equity include the inter-operability of data systems that allow the secure, free-flow of standardized data between stakeholders to minimize information asymmetry and improve decision making (further discussed in section digital technologies) and the early investment of governments and NGOs, particularly in low-income countries, for facilitating the broad-scale adoption of digital agriculture technologies [see (2, 39, 42) as examples].

## Digital technologies

Digital applications for better understanding animal welfare and productivity centre on sensors, across a variety of species and technologies (34). Video observation is applied in settings such as feed intake measurement for grazing cattle (43) and flocking behavior in housed poultry (44). These are applicable to the commodity livestock system type. Video-based automated assessment of direct animal health indicators are more applicable to value seekers, such as gait score measurement in poultry (45). Automated detection of specific ailments has employed more specialized optical capabilities in cameras, such as thermal analysis for poultry foot health (46), dermatitis in dairy cattle (47), and African Swine Fever (48).

Audio technology has also addressed animal health and welfare. Surveillance of coughing has been used in detection of respiratory-related wasting disease in pigs (49). Digital analysis of animal vocalizations has yielded management indicators of stress levels in housed chickens (50) and turkeys (51), grazing cattle (52), and stresses associated with ewe-lamb interactions (53). Feather pecking has been monitored by audio sensors (54), and analysis of pecking sounds has been used to measure feed intake and stress in chickens (55). Sensors that monitor odors (electronic nose) can detect animal health and welfare problems for housed poultry (56), and in diagnosing specific bacterial infections in cattle (57). These applications lend themselves to animal welfare surveillance in both commodity and value seeking systems by virtue of costs avoided, and the scale of operation available to offset costs per unit of production. It is notable that for commodity systems the surveillance element is both a cost incurred to assure market access and ethical considerations of animal production, whereas for value seekers the producer attaches consumer value to the product.

For extensive livestock systems, a large number of digital data collection processes are in use. Bahlo et al.'s (58) review identifies the need for decision tools which integrate this variety of data sources, along with the need for inter-operable data systems whereby secure, standardized data sets are shared between stakeholders for improved, evidence-based decision making. These authors identify public benefits arising from the enabling factors shown in the bottom row of Table 1, such that regional data sets are implemented for collaborative resource management; and private benefits in terms of animal productivity and welfare. Index-based drought insurance for East African livestock keepers based on satellite imagery and weather data provides one example (59), and feed supply monitoring with terrestrial sensors in Mongolia (60) another. Furthermore, Elsäßer et al. (42) set out applications of digital systems in Africa that cover farm management, finance, market access, the supply chain and broader macro market intelligence thereby demonstrating the ubiquitous nature of digital systems in a variety of livestock systems (i.e., subsistence, commodity, value-seeking and nature-based system). These require the data inter-operability listed amongst enabling factors in the right hand column of Table 1.

Avoidance or mitigation of disease for the provision of safe and ethically-produced food is offered by digitally enabled surveillance of causal and contributing factors. This has been demonstrated in both intensive animal production (61), and extensive grazing systems (62), and in environmental interactive systems such as tick-livestock relations associated with disease (63). Air quality inside livestock housing and pens is recognized as a contributor to reduced productivity as well as disease (64), and has been subject to control using digital air quality sensors and associated integration of software and hardware (65). This has included measurement of particulate matter and gaseous contaminants (66), and air flow in animal handling systems (67). These interventions address primarily risks and production costs, and existing business models have limited facility for generating consumer value from them. Having said that, no claims are being made herein about these tools being proven indicators of animal welfare; it is acknowledged that human observation of animal welfare remains critical (68). It is implicitly accepted that animal welfare measures are generally more intricate than one set of data on one particular trait and that welfare is a nuanced subject so it is ill-advised to claim good welfare from the results of a single technology. We do maintain however that incentives for management steps taken are strongly rooted in the generation of value along the supply chain, which in turn is enabled by information flows to the manager and onwards to the consumer.

While there is ample evidence of digital systems striving to enhance good animal welfare practice outcomes, sharing of data and data exchange platforms to support the One Health movement are under-explored in the literature. It is therefore recommended that production animals within the wider digital agriculture landscape, and the implications for human health outcomes, are considered in future inquiry.

## Conclusions

Digital technology has the potential to enable several important changes in livestock systems. Low-cost, field-robust and high precision sensors provide a substantial influx of cheap data to producers, and potentially onwards to processers, traders and consumers. Available data describes the location, movement and condition of animals, their feed, environment, health, the conditions influencing their welfare, and threats. Meanwhile other technology, like radio frequency identification or blue tooth tags, track, select and control the animal product as it passes through food systems, as per the demand signals from processors and consumers.

High dimensional modeling of multiple data streams offers ever more precise representation of complex food systems using machine learning and artificial intelligence where feasible [see of such technology provided by Fuentes et al. (69)]. As examples, chicken meat systems integrate feed, shed conditions, animal performance and labor availability to automate controllers and optimize such systems as data accumulates. In the case of block chain technology which takes the data along the supply chain, data are shared and stored via distributed cloud systems to enable access to consumers, producers and processors. Such systems are particularly relevant to complex animal production systems, especially those which target specific value opportunities or which face complex problems relating to health, welfare or biosecurity.

The ideal result of adoption is a substantial diversification of supply chains, increased monetization of animal product quality and more sensitive management to meet customer demands and environmental threats. Such changes have a significance beyond the immediate value generated because they indicate endogenous growth in livestock systems: the capacity of actors within livestock systems to manage livestock. We argue that these capacities are evolving along distinct pathways for the different livestock types.

We show a diversity of adoption patterns that conform to a range of economic models as a measure of knowledge to indicate endogenous growth (70). The growth of knowledge that accompanies the technology confers a growing ability to identify, confirm and respond to opportunities and threats in complex livestock systems.

Adoption of digital technologies in animal production systems is likely to expand. Prior to 2015, the term digital agriculture barely appeared. Its growth since then has created a corpus of interest amongst innovators, researchers and investors that requires, as we state in Cook et al. (7), organization according to a food system framework. The scope for the type of endogenous growth demonstrated in Table 1 seems boundless, as the system evolves to meet demands on multiple fronts (71). Adoption is likely to proceed in all four pathways described by Cook et al. [(7). p. 6]: data, control, modeling, and networking. Currently value-driven innovation appears to be ahead of discipline-based research which could support it. We see a bright future for equitable digital systems that create value by managing and reporting animal welfare throughout global agri-food supply chains. We expect future publications will report on advances in these technologies with a focus on sustainability of livestock systems that include digital systems for managing animal welfare and the advantages it brings to the value of livestock and their associated products.

## Data availability statement

The original contributions presented in the study are included in the article/reference list, further inquiries can be directed to the corresponding author/s.

## Author contributions

All authors listed have made a substantial, direct, and intellectual contribution to the work and approved it for publication.

## Funding

Funding contributing to this work is gratefully acknowledged from The World Bank's project—Digital Acceleration of Agricultural Transformation Study: Focus on the Downstream Segment of Agricultural Value Chains, and from Agrifutures and Food Agility CRC project J-011566-Co-designed Scoping Study to Unlock the Power of Digital.

## Conflict of interest

The authors declare that the research was conducted in the absence of any commercial or financial relationships that could be construed as a potential conflict of interest.

## Publisher's note

All claims expressed in this article are solely those of the authors and do not necessarily represent those of their affiliated organizations, or those of the publisher, the editors and the reviewers. Any product that may be evaluated in this article, or claim that may be made by its manufacturer, is not guaranteed or endorsed by the publisher.
